# The Effect of Service Learning on the Development of Trait Emotional Intelligence and Adversity Quotient in Youths: An Experimental Study

**DOI:** 10.3390/ijerph20064677

**Published:** 2023-03-07

**Authors:** Hok-Ko Pong, Paul Lam

**Affiliations:** 1 Faculty of Management and Hospitality, Technological and Higher Education Institute of Hong Kong, Hong Kong, China; 2 Department of Accountancy, Economics and Finance, Hong Kong Baptist University, Hong Kong, China

**Keywords:** service learning, emotional intelligence, adversity quotient, university students, experiential learning

## Abstract

This study aims to determine the effect of service learning (SL) on the trait emotional intelligence and adversity quotient of Chinese undergraduate students in Hong Kong. The SL programme lasted six months (at least 80 service hours). In a pre-test–post-test experimental design, students who participated in the SL during that time period were classified as the experimental group (*n* = 139; 69 male, 70 female), whereas students who never participated in SL were classified as the comparison group (*n* = 133; 66 male, 67 female). Both groups of participants were asked to finish the Wong and Law Emotional Intelligence Scale (WLEIS) and the Adversity Response Profile^®^ (ARP) before and after the SL programme. The results showed that there were no significant differences in WLEIS and ARP scores (at the pre-test) between the experimental and comparison groups. The results further revealed that students in the experimental group had better improvements in WLEIS and ARP than those in the comparison group after they completed SL. These findings provide valuable insights and implications for incorporating components of SL into interventions for youths to improve their ability to process emotions and overcome adversity.

## 1. Introduction

The development of trait emotional intelligence (EI) and adversity quotient (AQ) is the golden age in adolescence and early adulthood [1]. The level of an individual’s EI and AQ highly depends on life experiences. Charbonneau and Nicol [2] believed that, after entering adolescence, adolescents are in the stage of self-image shaping. At this stage, they are greatly affected by the surrounding environment. When a gap exists between the imaginary self and the self in the eyes of others, young people will constantly reflect on themselves and struggle with themselves [3]. Inner contests trigger several emotional reactions.

There is a negative association between emotional development and undesirable behaviours, such as substance abuse and violence [4]; therefore fostering youths’ emotional development is a worthy and necessary goal of education. However, the cultivation and development of trait emotional intelligence could prevent these dangerous behaviours caused by improper emotional management. For example, EI could be incorporated into the curriculum of schools [5]. Especially during adolescence, EI is highly associated with rapid cognitive growth [6].

However, higher education tends to focus on the development of students’ intelligence and cognitive abilities, rather than the cultivation of trait emotional intelligence and adversity quotient. The lack of appropriate emotional responses and negative learning experiences hinders the subsequent development of self-confidence, endurance, resilience [7], communication skills and emotional management [8]. Moreover, youths are in the key stage to consolidate emotional intelligence and adversity quotient.

Experiential learning, which includes service learning (SL) and work-integrated learning, has become increasingly popular in higher education [9]. These new and innovative pedagogies could provide opportunities and occasions for students to develop their trait emotional intelligence [10] and adversity quotient [9].

EI through SL has been promoted and studied in higher education in the West [11]. However, studies on the impact of SL on university students’ trait emotional intelligence in the Asia-Pacific region are rare [12]. In addition, SL has not yet been formally explored as a possible intervention that develops adversity quotient. Accordingly, this study addressed the following research questions:

Research Question 1: Would Chinese undergraduate students’ trait emotional intelligence (including self-emotion appraisal [SEA], other people’s emotion appraisal [OEA], regulation of emotion [ROE] and use of emotion [UOE]) improve by the intervention programme (SL) compared to students who never participated the SL programme?

Research Question 2: Would Chinese undergraduate students get better in their adversity quotient (including control, origin and ownership, reach and endurance) through the intervention programme (SL) compared with students who never participated in the SL programme?

### 1.1. Emotional Intelligence and Trait Emotional Intelligence

Intelligence is a broad concept. It not only refers to cognition, but also emotions [13]. The emotional quotient is part of intelligence theories [14]. Goleman [4] proposed the characteristics that comprise EI, namely self-awareness, self-regulation, motivation, empathy and social skills. Brotheridge [15] also introduced four factors that could be used to guide students’ EI development in practice: self-awareness, interpersonal relationships, adaptability and self-realisation.

There is a theoretical difference between trait EI (i.e., emotional self-efficiency) and ability EI (i.e., cognitive-emotional capability) [14]. Trait EI assessed by self-report questionnaires does not necessarily connect directly with general cognitive capability or its proxy measures. As an alternative, the ability EI measured by maximal performance testing is clearly correlated with such measures of cognitive ability. Therefore, ability emotional intelligence is a wide-ranging capability as it comprises the cognitive processing of emotions and emotional information. In the study, only the trait emotional intelligence is concerned.

Mayer and Salovey [14,16] developed and validated a self-report instrument for the measurement of trait emotional intelligence. Wong and Law [17] further recognised that the instrument had a strong four-factor model (SEA, OEA, ROE and UOE) and was particularly suitable in the Chinese sample; hence, they developed the Wong and Law Emotional Intelligence Scale (WLEIS) [17]. In this study, the WLEIS [17] was employed to assess the youths’ trait emotional intelligence. It includes SEA, OEA, ROE and UOE.

Self-emotion appraisal refers to an individual’s capability to recognise and understand what they are feeling and express emotions as intended. Other people’s emotion appraisal refers to the capability to comprehend, perceive and identify the emotions of others. Regulation of emotion refers to the capability to monitor, assess and perform to adjust feelings. Use of emotion refers to the capability to utilize emotions, such as turning emotions into positive actions and better performance. 

Elias and Moceri [18] believed that social and emotional learning plays an important role in the education system. It refers to the efforts to develop character and civic education, language skills and emotional intelligence in schools. It involves teaching tools designed to help students learn skills and successfully manage life tasks [19]. Lewkowicz (2007) recognised the importance of integrating emotional intelligence into the teaching process. Hence, Lewkowicz [20] emphasised curriculum-based activities to cultivate students’ trait emotional intelligence. Researchers [21,22] have shown that activities and curricula that promote the emotional development of youths are needed in school environments.

### 1.2. Adversity Quotient

Adversity means an unfortunate event or situation or a state of serious and persistent difficulty [23]. Adversity quotient (AQ) is a measure of how individuals perceive and respond to difficulties and adversity [24]. Moreover, the level of happiness and cheerfulness of individuals always depends on their ability to overcome adversity [25].

Stoltz [24] developed the adversity quotient (AQ) theory, which is a reflection of how people face adversity according to the concepts of three sciences—cognitive psychology, mental and neurophysiological neuroimmunology. This type of reaction is called a combination of adversity quotient and emotional intelligence (EI), and this is the natural response of human instinct in adversity.

In this study, the Adversity Response Profile^®^ (ARP) developed by Stoltz [24] was utilised to assess the ability of individuals to respond to adversity. Adversity quotient consists of four major dimensions, referred to as CO2RE (control, origin and ownership, reach and endurance).

Control refers to the ability to perceive the possibility of changing a situation. It measures how well a person thinks he/she has control over adverse events. It is a powerful indicator of resilience and health.

On the one hand, origin asks whether adversity and frustration are caused by someone or something and then how much responsibility a person should have for adversity and setbacks. On the other hand, ownership means the degree of willingness to take responsibility for improving a situation. It is a powerful indicator of responsibility and the possibility of action.

Reach refers to the perception of how far a difficulty extends into other domains of life. It is a powerful indicator of burden and stress levels. Moreover, it indicates how far adversity and frustration go into other areas of life.

Endurance means the perceived length of time adversity will last [24]. It looks at two questions: (1) how long adversity and frustration may endure (2) and how long the consequences of adversity and setbacks may last. It is a strong measure of hope or optimism.

Schools play an important role managing and providing student activities that can help them develop their adversity quotient [7]. Devakumar [26] showed that students from different types of schools differ in their AQ scores. The results further indicated that AQ, school atmosphere and school performance correlate with one another. Devakumar [26] further indicated that AQ could be integrated into schools’ curriculum and other development programmes. This strategy serves as an improvement in communication and relationship between students, teachers and principals.

### 1.3. Service Learning and Its Relationship with the Traits EI and AQ

Service learning is a form of experiential learning and it consists of two key components: service and learning [27]. Service learning is beneficial to the balanced development of children and reached holistic learning in schools [28]. Service learning is not only considered an effective teaching tool but also a good tool to connect students with the community [29]; it is a tool through which colleges and universities can bring positive changes to society [12]. Moreover, SL enriches the insights and skill development of students’ personal and interpersonal emotional intelligence and adversity ability. For example, McKinnon and Fealy [30] mentioned the seven Cs of successful SL, including compassion, curiosity, courage, collaboration, creativity, capacity building and competence. Service learning programmes encourage young people to broaden their horizons, enrich life experience and accelerate growth and maturity [31].

Service learning is associated with academic objectives in the curriculum, student achievement and socio-emotional growth and responses to difficulties [32]. For example, SL can improve test scores and student attendance as well as build a harmonious school atmosphere and decrease the number of disciplinary actions [33]. Keating et al. [34] introduced emotional intelligence into the curriculum, and multiple management skills courses were successfully used to improve students’ EI [35,36].

Incorporating SL into curricula and connecting the components of emotional intelligence [37] and adversity intelligence [26] can improve the effectiveness of schools’ ability to teach professionalism and communication skills, and ensure that best practices and methods are in place for industry-wide applications [37].

To be authentic, these learning experiences are always grounded in reflective practices, which help students connect to core content knowledge and its real-world applications [12]. Emotional intelligence [37] and adversity intelligence [26] have been considered important parts of the education process. The exploratory study conducted by Manring [38] found that students’ SL experience cultivates personal and social aspects of EI.

### 1.4. Present Study

The objective of the current study was to examine if a SL programme at a university in Hong Kong would be useful and positive in boosting students’ trait emotional intelligence and adversity quotient. For example, the service experiences offered to the community include help and support to disadvantaged community members. The measure of trait emotional intelligence and the measure of adversity quotient were administered at pre-test and post-test.

## 2. Method

### 2.1. Participants

A total of 315 undergraduate students from the same university joined in the pre-test, and 272 of those undergraduate students also joined in the post-test. Of these 272 participants who joined in both the pre-test and post-test, 139 participants completed the SL programme during that period. These 139 participants served as the experimental group. The remaining 133 participants who had never attended programmes associated with SL served as the comparison group. Therefore, 139 participants in the experimental group (*M* age = 23.34, *SD* = 0.99; 69 male: 49.6%, 70 female: 50.4%) and 133 participants in the comparison group (*M* age = 23.28, *SD* = 1.18; 66 male: 49.6%, 67 female: 50.4%) were included and identified in the study. No group differences by age and gender were observed. The results shown in Table 1 and Table 2 showed that these two groups of participants had roughly the same background in emotional intelligence and adversity quotients.

### 2.2. Measures

There were two versions of the questionnaires, and participants could complete either the Chinese or English version. The measures (WLEIS and ARP) were translated as necessary so that both a Chinese version and an English version were available.

### 2.3. Demographics

Participants gave demographic data, including gender, age, years in university (e.g., Year 3 students), length of part-time work experience and related income.

### 2.4. Emotional Intelligence: Wong and Law Emotional Intelligence Scale (WLEIS)

On the WLEIS [17], participants rated how much they accepted each of 16 question items, using a five-point Likert scale (1 = strongly disagree to 5 = strongly agree). The scale was used to assess the four areas of emotional intelligence:

1. Self-emotion appraisal (SEA; 4 items, e.g., “I have a good sense of why I have certain feelings most of the time”);
2. Others’ emotion appraisal (OEA; 4 items, e.g., “I always know my friends’ emotions from their behaviour”);
3. Use of emotion (UOE; 4 items, e.g., “I am a self-motivated person”);
4. Regulation of emotion (ROE; 4 items, e.g., “I am able to control my temper and handle difficulties rationally”).

The scores for the overall and the subscale (on specific dimensions) are computed and presented as means. In the pre-test results of this study, acceptable internal consistency reliabilities were obtained: SEA (0.941), OEA (0.954), UOE (0.976), ROE (0.982) and overall emotional intelligence scale (0.736). Exploratory factor analysis identified four factors (each with an eigenvalue > 1.0) corresponding to the ROE, UOE, OEA and SEA dimensions, which explained 27.053%, 24.005%, 21.531% and 18.583% of the variance, respectively. Principal component analysis (PCA) revealed that the four-dimension model was suitable and applicable for use in our sample (Kaiser–Meyer–Olkin value = 0.900; Bartlett’s test of sphericity significant at *p* < 0.001). Table 3 reveals the factor loadings for the four-dimension model of the pre-test and post-test.

### 2.5. The Adversity Response Profile

On the ARP [24], students ranked how much they feel in each of 20 situations, using a five-point Likert scale (1 = not at all to 5 = completely). This measure was utilized to measure four aspects of adversity quotient:

1. Control (5 items, e.g., “Your personal and work obligations are out of balance. To what extent can you influence this situation?”);
2. Origin and ownership (5 items, e.g., “Your workplace is understaffed. To what extent do you feel responsible for improving this situation?”);
3. Reach (5 items, e.g., “You missed an important appointment. To what extent do you feel that the consequences of this situation will affect you?”);
4. Endurance (5 items, e.g., “After an extensive search, you cannot find an important document. What are the consequences of this situation?*”*).

In the pre-test results of this study, acceptable internal consistency reliabilities were obtained: control (0.816), origin and ownership (0.955), reach (0.913), endurance (0.900) and overall Adversity Response Profile (0.905). Exploratory factor analysis identified four factors (each with an eigenvalue > 1.0) corresponding to origin and ownership, reach, endurance and control domains, which explained 36.565%, 16.058%, 13.658% and 8.027% of the variance, respectively. Principal component analysis revealed that the four-domain form was suitable and applicable for our sample (Kaiser–Meyer–Olkin value = 0.851; Bartlett’s test of sphericity significant at *p* < 0.001). Table 4 displays the factor loadings for the four-factor model, including the pre-test and post-test in the current sample. Both scales (WLEIS) and (ARP) demonstrated satisfactory internal consistencies at the pre-test and post-test.

### 2.6. Procedure

Ethical consent for this research was obtained from the research ethics committee of the authors’ university. The aim of the research was explained to the participants, and they provided verbal and written consent to participate in the research. The research setting was a university in Hong Kong, and the sampling methodologies used were convenience and snowballing sampling. The target population was baccalaureate business students, and the data collection ran for 16 weeks.

The SL programme was actively introduced, promoted and supported by the teachers and administrators of the university’s Business School. The pre-test questionnaires were self-filled from March to May 2021. Participants were given paper-and-pencil questionnaires in class, and after completing the questionnaires, participants returned them to the course instructor. Each participant spent around 15 min to fill out the questionnaire. Non-participating students and students who had enrolled in an SL programme previously were exempted. Thus, the students who participated in SL at that period were classified as the experimental group, whereas students who had never enrolled in SL were classified as the comparison group. Lastly, 291 out of 410 invited participants completed the questionnaires, with a response rate of 71%.

The post-test was performed as a continuation and subsequent assessment (after the SL programme, i.e., completion of the intervention) of the 291 participants. It took place between 6 and 15 September 2021. Both questionnaires were again self-filled and also the questionnaires were sent to the same 291 participants. Ultimately, data from 272 participants were successfully traced and matched between the pre-test and post-test. The attrition rate of 6.52% was satisfactory compared to other longitudinal studies [28]. There were no significant variances in gender, age, EI and AQ scores between the matched sample (*n* = 272) and participants who withdrew from the study (*n* = 19).

Participants provided written informed consent. They were informed of the research purpose and that their participation was voluntary. Moreover, they were free to withdraw at any point during the research without consequence, and no reward was provided.

The front page of the questionnaire indicated that all data collected would be kept for statistical purposes only, and their answers would remain strictly confidential. Although participants replied with their answers anonymously, they were requested to write the same code number (e.g., a number that they could easily remember) on each reply. By doing so, replies from the same participant collected at the start and completion of the SL programme could be matched. The details, including instructions and guidance, were given at the opening of the questionnaire.

### 2.7. Intervention: Service Learning (SL)

The SL programme is one of the General Education elective modules, offered to Year 2 and Year 3 full-time undergraduate students. The beneficiaries of the programme include vulnerable groups such as seriously sick or recovering patients and their families, hearing-impaired students and students with learning disabilities. They were assigned similar service content, and there was no systematic difference between the community services assigned. Aside from getting acquainted with the local neighbourhood and vulnerable groups, SL participants were able to apply the knowledge and techniques they learned in class to develop their aptitudes through a series of SL tasks. Ultimately, this project could help university students develop their trait emotional intelligence and adversarial quotient.

The duration of the SL programme was 6 months, and it had three phases: (1) Preparation: March–June, (2) Execution of assigned tasks: June–August (80 h minimum) and (3) Feedback and review: September. In pre-service, the pre-employment preparation and workshops were conducted (4 lessons: 2 h 45 min each). Subsequently, students were divided into a group of four. During the service phase, the course teachers and organisation staff supervised and advised students at a 1:4 ratio. Each service group was required to meet with the course teacher six times (3 h each) for the duration of the community service. In these meetings, the course teacher provided supervision and support for the SL activities, such as psychological counselling and resource provision. In the post-service period, participants gave an oral presentation and submitted a reflective essay. The supervisors facilitated students’ reflection by raising questions such as what were the positive outcomes of the activities and what changes they recognized in their lives or perceptions.

On the one hand, students who participated in SL considered SL an opportunity to support and help students with intellectual disabilities. On the other hand, recipients were happy to know that someone cared about them. Groups of four students would provide day services in the school’s dormitory. Services included training and support for students with learning disabilities. At the start, the service period was scheduled to run from early July to late August 2021. However, students were requested to assist in the residence halls in late June so that they could be familiarized with the residence hall students. Students were also asked to submit their activity proposals in early July.

## 3. Results

Data analysis was performed using SPSS Version 27 (IBM Corp., Armonk, NY, USA), with no loss of data. The necessary steps were performed, such as data cleaning to correct coding errors and illogical data values (if required).

### 3.1. Descriptive Statistics

Table 1 and Table 2 show the descriptive figures and the results of assessments examining possible demographic variances in emotional intelligence (including SEA, OEA, ROE, UOE and overall score) and adversity quotient (including control, origin and ownership, reach, endurance and overall score). A series of *t*-tests and one-way analyses of variance (ANOVAs) were conducted to compare different levels of each demographic variable on the WLEIS and ARP scores. These analyses were conducted separately for the pre-test and post-test scores. There are no significant differences in WLEIS and ARP scores (pre-test and post-test) between the two groups in age (*p* > 0.05), according to one-way analyses of variance (ANOVAs). Also, there were no significant differences in WLEIS and ARP scores (pre-test and post-test) between the two groups in gender and years of study (*p* > 0.05), according to *t*-test.

### 3.2. The Effects of SL Programme on Chinese University Students’ EI and AQ

The first set of analyses was performed to confirm the impact of the SL programme on students’ EI and AQ. In the pre-test, eight independent *t*-tests indicated that there were no statistically substantial differences in EI and AQ scores between the experimental and comparison groups before the service-learning activities. Nevertheless, at the post-test, there were statistically significant differences in EI scores (including SEA, OEA, UOE, ROE and overall score) and AQ scores (including control, origin and ownership, reach, endurance and overall score) between the two groups after the intervention programme. Table 5 shows the results.

A sequence of mixed between-within ANOVAs was executed to contrast the two different groups on the pre-test and post-test evaluations of participants’ EI and AQ scores. In the following analysis, the within-subject factor was set as Period (before and after the intervention: SL, i.e., pre-test and post-test), the between-subject factor was fixed as Group (experimental and comparison) and the dependent variables were the four dimensions of EI and the four domains of AQ. Table 5 displays all dimensions of EI and AQ exhibited significant Period × Group interaction effects, indicating that over time these two groups held different perceptions of these features. By contrast, the results showed no group differences at pre-test, and the experimental group had better performance and higher marks on all scales at post-test.

Table 5 displays a significant interaction between Group and Time (Period). The interaction effects on SEA, *F* (1270) = 732.472, *p* < 0.001; OEA, *F* (1270) = 611.942, *p* < 0.001; UOE, *F* (1270) = 569.308, *p* < 0.001 and ROE, *F* (1270) = 649.270, *p* < 0.001 of EI, and control, *F* (1270) = 31.549, *p* < 0.001; origin and ownership, *F* (1270) = 26.537, *p* < 0.001; reach, *F* (1270) = 31.452, *p* < 0.001 and endurance, *F* (1270) = 28.724, *p* < 0.001 of AQ kept significant when age and gender were controlled in the analyses. Post-hoc comparisons exhibited that the experimental group had higher marks for SEA, OEA, UOE and ROE of EI as well as for control, origin and ownership, reach and endurance of AQ at post-tests but not at pre-tests.

Table 5 displays that these effects are qualified by a significant Time × Group interaction for EI (including SEA, OEA, UOE and ROE) as well as AQ (including control, origin and ownership, reach and endurance). These interactions revealed that between the repeated assessments, youths’ mean score variations on all specific dimensions of EI and AQ varied with whether they were in the experimental group (i.e., whether they participated in SL).

## 4. Discussion

In this study, we examined whether Chinese university students’ trait emotional intelligence and adversity quotient would be altered owing to being cultivated through community service learning. The experimental design compared a group of students enrolled in a SL class (experimental group) and another group of students who were not enrolled in the class (control condition). The comparison was based on their emotional intelligence and adversity quotient before and after the experiment. The results showed that SL promoted the development of trait emotional intelligence and adversity quotient among Chinese university business students. The study found that students’ reflection in the programme is most effective and beneficial for youths’ development in dealing with emotions and adversity. It is because the participants had the opportunity to reflect on the entire process of the SL programme, and they were also asked to present what they found during the SL experience.

### 4.1. The Impact of Community Service Learning on Emotional Intelligence

Martinez [39] and Billig [33] found that incorporating SL as a teaching strategy into the curriculum provides a learning experience to improve university students’ trait emotional intelligence. The findings of the current study on SL as a process of students’ emotional growth are supported by the studies of Billig [33] and Elias [40]. Our findings were backed by Sharifi et al. [41], who demonstrated the importance of the process of reflecting on SL experiences for gaining insight into students’ emotions and behaviour. The findings indicated that SL contributed to students’ emotional intelligence, including SEA, OEA, ROE and UOE.

The outcomes of the current research are consistent with the study of Martinez [39], who found significant positive correlations between SL and introspection emotional quotient. Similarly, Eyler and Giles [42] and Moely et al. [43] demonstrated that SL had a positive effect on students’ personal development, including SEA. Moreover, SL could positively affect cognitive, affective and psychomotor development, which are functions of self-emotion judgment [44]. Our findings were supported by Hanna and Treece [5], who indicated an improvement in students’ self-awareness and self-management, which is closely linked with self-emotion judgment through SL.

Our findings were consistent with the findings of Martinez [39], who showed significant positive correlations between SL and interpersonal emotional quotient. Our findings were supported by Hanna and Treece [5], who found that, through SL, students improved social awareness and social skills, especially empathy, which are closely linked with other people’s emotion appraisal. In addition, Eyler and Giles [42] and Moely et al. [43] found that SL had a positive effect on students’ interpersonal development, such as an increase in interest in civic and community issues and diversity attitudes, which favoured the development of trait emotional intelligence and adversity quotient in youths.

Manring [38] pointed out that SL offered a meaningful experience that transforms students’ perspectives into other-centred views, together with the self-reflection process promoted by teachers, which helps develop skills and behaviour linked with other people’s emotion assessment. In addition, a strong and meaningful programme, such as SL, requires community participation, multi-levels of interpersonal interaction and clear communication and interdisciplinary teamwork, which greatly reinforce the roles of other people’s emotion appraisal [39].

Our findings are consistent with the research of Astin and Sax [31], who found a substantially close relationship between SL and the management of emotional feelings. Strayhorn (2008) indicated that SL has positive impacts on nurturing students’ self-discipline through changes in their behaviour, habits and attitudes, because students’ day-to-day life can be improved through SL activities. Besides, the studies found that SL is an effective teaching method to improve introspection and self-control [31,45,46].

Our findings are consistent with those of Manring [38], who indicated that SL experience has a great impact on students’ emotional self-motivation. Participation in such programmes has been shown to strengthen leadership [43], where the use of emotions is enhanced. In addition, SL cultivates students’ self-efficacy and self-confidence [31,46,47], which facilitates the proper use of their emotions.

### 4.2. The Impact of Community Service Learning on the Development of Adversity Quotient

Hamner et al. [44] and Garmezy et al. [47] also found that SL positively contributes to the development of university students’ adversity quotient, including control, origin and ownership, reach and endurance.

The studies revealed that, through SL, students gradually improved their problem-solving and decision-making skills [48], autonomy, confidence [49] and leadership skills [43]. These characteristics and features are closely linked with the control domain of AQ. Moreover, Conway et al. [50] and Novak et al. [51] found a close positive correlation between AQ and performance, such as academic success and professional achievement. These improved capabilities help individuals control adverse events and enhance their awareness of the possibility of environmental changes.

Our research results are consistent with the finding of Godfrey et al. [52] that SL has a significant impact on students’ civic obligations and social concerns, because students are trained to be responsible through different tasks assigned in community services. Specifically, SL has a significant impact on students’ sense of mission and teamwork spirit, which are related to the positive and constructive response of AQ in terms of origin and ownership [53].

Responsibility does not mean excessive blame and perfectionism. Adversity quotient emphasises the balance between the two [24]. By participating in SL, students can develop good personalities and character, such as a higher level of psychological maturity [45], serious work attitude [29], proactive attitude and commitment to others [54].

This finding is consistent with Hulaikah and Degeng’s research [9], which shows that SL helps students build the ability to adapt to burdens and stress. By participating in these community service activities including volunteer and charity work, students can get to know people from all walks of life and learn to look at difficulties from another perspective. Mellor et al. [55] have shown that volunteering and charity work cultivate positive thinking and optimistic attitudes among young people. Hulaikah and Degeng [9] found that SL students develop tolerance and are less stubborn. Moreover, Hulaikah and Degeng [9] and Rhoads [56] found that SL activities help students resolve problems and difficulties on their own, and then analyse problems objectively to avoid reaching a dead end and giving up. Our findings were supported by Garmezy et al. [47], who found that SL students can develop protective factors that enable individuals to abandon maladaptive behaviours and adopt constructive and beneficial responses to stressors.

Our findings were supported by Hamner et al. [44], who indicated that SL cultivates students’ perseverance, patience and determination, because students need to provide services to the community on a regular basis, including charities, hospitals and schools. These projects usually take several months and require perseverance, courage and determination to complete. Therefore, this series of experiences can cultivate the regularity and discipline of their kindness to society. Participants learn to tolerate and accept others. These actions are a process of cultivating perseverance and patience.

### 4.3. Limitations

This research has four limitations. Firstly, the sample of 272 participants from one major in one university is smaller than the entire population; hence, the generalisability of the findings may be limited. In future research, more samples should be recruited to attain better representativeness.

Secondly, the study data were obtained from self-report questionnaires. Hence, differences in sharing methods may have caused exaggerated effects. In addition, participants may not objectively reply to their own trait emotional intelligence or adversity quotient accurately, and the measures do not include a validity measure for detecting inconsistent responses or social expectations. Thus, future research can explore using behavioural indicators of these constructs, ratings provided by course teachers and classmates and feedback from programme beneficiaries to gain insights into the effectiveness of SL.

Thirdly, the conclusions are based on students from only one SL course for Chinese business students in Hong Kong. Thus, future researchers are encouraged to examine the generalisability of these findings to other types of SL, students and sociocultural settings.

Lastly, This SL program is relatively short, but the improvements are surprising. In fact, the roles of teachers, families, and peer groups are very important factors affecting the emotional development of adolescents. More objective indicators, such as family background and support from schools and teachers, should be considered in the future.

## 5. Conclusions

The study offers ideas and direction for professionals in higher education interested in service-learning programmes as a tool to improve university students’ trait emotional intelligence and adversity quotient. The findings of the study indicate that undergraduate students demonstrated improvements in these areas after participating in a 6-month SL programme. The outcomes are consistent with Wang et al.’s [57] evidence of the positive effects of SL on Chinese undergraduate students. Furthermore, this study is the first study on the effect of SL on the trait emotional intelligence and adversity quotient of business undergraduate students in Asia. The results have valuable implications for incorporating and strengthening the components of SL into interventions for youths to boost their abilities in handling emotions and overcoming adversities.

## Figures and Tables

**Table 1:** Descriptive Statistics: Participants’ Demographics and their Relationship with Emotional Intelligence Scale (WLEIS).

Factors	N (%)	Pre-Test					Post-Test				
		SEA	OEA	UOE	ROE	Overall	SEA	OEA	UOE	ROE	Overall
All		3.18 (0.42)	3.00 (0.39)	2.22 (0.62)	3.20 (0.69)	2.90 (0.26)	3.68 (0.53)	3.49 (0.55)	2.74 (0.74)	3.68 (0.81)	3.40 (0.50)
Groups											
Experiment Group	139 (51.1%)	3.12 (0.36)	3.00 (0.38)	2.25 (0.65)	3.14 (0.71)	2.88 (0.24)	4.04 (0.28)	3.89 (0.28)	3.15 (0.26)	4.04 (0.75)	3.78 (0.26)
Control Group	133 (48.9%)	3.25 (0.46)	2.99 (0.40)	2.19 (0.58)	3.25 (0.68)	2.92 (0.27)	3.30 (0.46)	3.07 (0.45)	2.30 (0.59)	3.31 (0.69)	2.99 (0.35)
		t = −2.64	t = 0.04	t = 0.05	t = −1.31	t = −1.42	t = 16.02 ***	t = 18.27 ***	t = 11.65 ***	t = 8.28 ***	t = 20.96 ***
Gender											
Male	135 (49.6%)	3.17 (0.42)	2.99 (0.44)	2.26 (0.66)	3.25 (0.65)	2.92 (0.24)	3.64 (0.51)	3.44 (0.58)	2.76 (0.76)	3.72 (0.78)	3.39 (0.50)
Female	137 (50.4%)	3.20 (0.42)	3.00 (0.33)	2.18 (0.57)	3.15 (0.73)	2.88 (0.27)	3.71 (0.54)	3.53 (0.52)	2.71 (0.72)	3.65 (0.84)	3.40 (0.50)
		t = −0.53	t = −0.04	t = 1.00	t = 1.20	t = 1.18	t = −1.11	t = −1.43	t = 0.59	t = 0.74	t = −0.17
Age											
21	6 (2.2%)	3.33 (0.52)	3.00 (0.01)	2.42 (0.49)	3.17 (0.75)	2.98 (0.26)	3.38 (0.49)	3.21 (0.40)	2.63 (0.77)	3.38 (0.74)	3.15 (0.47)
22	66 (24.3%)	3.21 (0.45)	3.06 (0.43)	2.20 (0.60)	3.25 (0.66)	2.93 (0.26)	3.63 (0.47)	3.48 (0.55)	2.68 (0.74)	3.67 (0.81)	3.36 (0.49)
23	85 (31.3%)	3.19 (0.41)	2.99 (0.36)	2.19 (0.59)	3.10 (0.67)	2.87 (0.21)	3.76 (0.56)	3.53 (0.56)	2.78 (0.70)	3.64 (0.80)	3.35 (0.48)
24	68 (25%)	3.11 (0.36)	2.98 (0.31)	2.32 (0.64)	3.24 (0.74)	2.91 (0.26)	3.66 (0.48)	3.68 (0.50)	2.85 (0.77)	3.79 (0.83)	3.47 (0.51)
25	47 (17.3%)	3.21 (0.47)	2.95 (0.50)	2.13 (0.67)	3.23 (0.70)	2.88 (0.30)	3.66 (0.60)	3.35 (0.62)	2.59 (0.77)	3.66 (0.82)	3.31 (0.52)
		*F*(4267) = 0.79	*F*(4267) = 0.69	*F*(4267) = 0.86	*F*(4267) = 0.60	*F*(4267) = 0.86	*F*(4267) = 1.15	*F*(4267) = 1.54	*F*(4267) = 1.10	*F*(4267) = 0.62	*F*(4267) = 1.18
Year of studies											
Year 2	150 (55.1%)	3.19 (0.39)	23.00 (0.34)	2.26 (0.61)	3.20 (0.73)	2.91 (0.25)	3.89 (0.48)	3.62 (0.52)	2.86 (0.73)	3.79 (0.82)	3.51 (0.47)
Year 3	122 (44.9%)	3.17 (0.45)	2.99 (0.44)	2.17 (0.62)	3.20 (0.65)	2.88 (0.26)	3.55 (0.55)	3.32 (0.55)	2.59 (0.73)	3.56 (0.79)	3.25 (0.50)

Note. *N* = 272. *** *p* < 0.001.

**Table 2:** Descriptive Statistics: Participants’ Demographics and their Relationship with the Adversity Response Profile (ARP).

Factors	N (%)	Pre-Test					Post-Test				
		Control	Origin and Ownership	Reach	Endurance	Overall	Control	Origin and Ownership	Reach	Endurance	Overall
All		3.18 (0.50)	2.38 (0.90)	2.55 (0.74)	2.75 (0.58)	2.71 (0.58)	3.68 (0.69)	2.88 (1.01)	3.05 (0.82)	3.26 (0.78)	3.22 (0.68)
Groups											
Experiment Group	139 (51.1%)	3.12 (0.51)	2.35 (0.90)	2.53 (0.75)	2.76 (0.58)	2.69 (0.48)	3.78 (0.68)	3.01 (0.96)	3.21 (0.85)	3.42 (0.74)	3.36 (0.65)
Control Group	133 (48.9%)	3.24 (0.49)	2.41 (0.91)	2.56 (0.74)	2.75 (0.58)	2.74 (0.48)	3.58 (0.70)	2.75 (1.04)	2.89 (0.76)	3.09 (0.79)	3.08 (0.68)
		t = −2.016 *	t = −0.529	t = −0.284	t = 0.050	t = −0.865	t = 2.409 *	t = 2.186 *	t = 3.220 **	t = 3.513 **	t = 3.430 **
Gender											
Male	135 (49.6%)	3.15 (0.47)	2.36 (0.90)	2.51 (0.79)	2.67 (0.60)	2.67 (0.49)	3.66 (0.70)	2.88 (1.04)	3.03 (0.89)	3.18 (0.84)	3.19 (0.72)
Female	137 (50.4%)	3.21 (0.54)	2.39 (0.91)	2.58 (0.69)	2.83 (0.54)	2.76 (0.48)	3.71 (0.69)	2.89 (0.98)	3.08 (0.75)	3.33 (0.72)	3.25 (0.64)
		t = −1.014	t = −0.245	t = −0.842	t = −2.322 *	t = −1.393	t = −0.562	t = −0.098	t = −0.493	t = −1.543	t = −0.772
Age											
21	6 (2.2%)	3.53 (0.39)	2.57 (1.29)	2.87 (1.31)	2.60 (0.64)	2.89 (0.81)	3.70 (0.43)	2.73 (1.13)	2.97 (0.95)	2.77 (0.65)	3.04 (0.65)
22	66 (24.3%)	3.17 (0.45)	2.30 (0.89)	2.52 (0.71)	2.61 (0.52)	2.65 (0.44)	3.68 (0.64)	2.82 (0.99)	3.04 (0.81)	3.12 (0.76)	3.17 (0.65)
23	85 (31.3%)	3.14 (0.53)	2.46 (0.93)	2.51 (0.68)	2.77 (0.59)	2.72 (0.49)	3.69 (0.71)	3.01 (1.01)	3.08 (0.73)	3.33 (0.76)	3.28 (0.66)
24	68 (25%)	3.23 (0.53)	2.41 (0.87)	2.56 (0.73)	2.59 (0.59)	2.78 (0.48)	3.71 (0.69)	2.90 (1.01)	3.05 (0.81)	3.38 (0.80)	3.26 (0.69)
25	47 (17.3%)	3.16 (0.50)	2.26 (0.89)	2.59 (0.84)	2.74 (0.57)	2.69 (0.50)	3.63 (0.78)	2.72 (1.02)	3.04 (1.01)	3.20 (0.83)	3.15 (0.76)
		*F*(4267) = 1.080	*F*(4267) = 0.603	*F*(4267) = 0.394	*F*(4267) = 2.232	*F*(4267) = 0.800	*F*(4267) = 0.106	*F*(4267) = 0.756	*F*(4267) = 0.056	*F*(4267) = 1.718	*F*(4267) = 0.550
Year of studies											
Year 2	150 (55.1%)	3.19 (0.49)	2.37 (0.86)	2.58 (0.77)	2.74 (0.58)	2.72 (0.48)	3.69 (0.69)	2.87 (0.97)	3.08 (0.84)	3.24 (0.78)	3.22 (0.68)
Year 3	122 (44.9%)	3.17 (0.52)	2.39 (0.96)	2.51 (0.71)	2.77 (0.58)	2.71 (0.49)	3.68 (0.70)	2.89 (1.06)	3.02 (0.80)	3.28 (0.79)	3.22 (0.68)

* *p* < 0.05, ** *p* < 0.01.

**Table 3:** Results of Exploratory Factor Analysis of items on the Wong and Law Emotional Intelligence Scale WLEIS (N = 272).

	Pre-Test Results				Post-Test Results			
	ROE	UOE	OEA	SEA	ROE	UOE	OEA	SEA
Q1.	−0.045	−0.068	0.042	**0.915**	0.102	0.137	0.347	**0.861**
Q2.	−0.043	−0.010	0.030	**0.931**	0.112	0.167	0.220	**0.878**
Q3.	−0.060	−0.078	−0.080	**0.915**	0.156	0.169	0.259	**0.836**
Q4.	−0.040	0.034	−0.020	**0.929**	0.102	0.268	0.207	**0.819**
Q5.	0.004	0.014	**0.979**	−0.040	0.220	0.285	**0.851**	0.267
Q6.	−0.020	0.020	**0.968**	−0.045	0.270	0.281	**0.796**	0.345
Q7.	0.074	0.069	**0.947**	0.007	0.258	0.284	**0.819**	0.313
Q8.	0.109	−0.128	**0.870**	0.048	0.274	0.131	**0.818**	0.347
Q9.	−0.104	**0.954**	0.000	0.041	0.072	**0.906**	0.194	0.219
Q10.	−0.031	**0.946**	−0.025	−0.078	0.159	**0.899**	0.199	0.122
Q11.	−0.098	**0.975**	−0.009	−0.061	0.073	**0.916**	0.197	0.172
Q12.	0.024	**0.980**	0.007	−0.027	0.145	**0.918**	0.172	0.207
Q13.	**0.960**	−0.052	0.051	−0.116	**0.941**	0.107	0.212	0.044
Q14.	**0.982**	−0.053	0.024	−0.058	**0.949**	0.126	0.187	0.115
Q15.	**0.976**	−0.033	0.037	−0.050	**0.939**	0.118	0.200	0.122
Q16.	**0.965**	−0.071	0.061	0.023	**0.925**	0.093	0.190	0.192

Note: Items loaded on each factor are in boldface.

**Table 4:** Results of Exploratory Factor Analysis of items on the Adversity Response Profile (N = 272).

	Pre-Test Results				Post-Test Results			
	Origin and Ownership (O2)	Reach (R)	Endurance (E)	Control (C)	Origin and Ownership (O2)	Reach (R)	Endurance (E)	Control (C)
Q1.	0.139	0.127	0.241	**0.572**	0.218	0.246	0.367	**0.619**
Q2.	**0.889**	0.110	0.133	0.143	**0.866**	0.197	0.198	0.198
Q3.	0.095	**0.781**	0.006	0.178	0.137	**0.791**	0.099	0.304
Q4.	0.223	0.104	**0.498**	0.303	0.286	0.203	**0.560**	0.420
Q5.	0.052	**0.954**	0.003	0.096	0.107	**0.883**	0.070	0.263
Q6.	**0.885**	0.116	0.145	0.064	**0.869**	0.187	0.202	0.138
Q7.	0.206	0.133	0.047	**0.665**	0.265	0.269	0.197	**0.675**
Q8.	0.143	−0.011	**0.915**	0.221	0.229	0.092	**0.858**	0.353
Q9.	0.113	**0.955**	0.033	0.130	0.157	**0.875**	0.081	0.268
Q10.	0.164	−0.028	**0.670**	0.212	0.255	0.146	**0.690**	0.386
Q11.	**0.865**	0.137	0.234	0.150	**0.841**	0.193	0.269	0.227
Q12.	0.112	**0.947**	0.041	0.149	0.400	**0.667**	0.409	0.053
Q13.	0.092	0.085	0.155	**0.682**	0.184	0.208	0.282	**0.704**
Q14.	0.129	0.082	**0.952**	0.156	0.213	0.179	**0.886**	0.298
Q15.	0.067	0.094	0.255	**0.850**	0.182	0.186	0.347	**0.820**
Q16.	**0.904**	0.073	0.168	0.132	**0.886**	0.140	0.218	0.202
Q17.	0.041	0.133	0.232	**0.841**	0.161	0.212	0.325	**0.820**
Q18.	**0.907**	0.095	0.089	0.162	**0.894**	0.159	0.147	0.218
Q19.	0.155	0.075	**0.933**	0.180	0.237	0.178	**0.867**	0.322
Q20.	0.400	**0.487**	0.222	0.046	0.400	**0.667**	0.413	0.065

Note: Items loaded on each factor are in boldface.

**Table 5:** The Main Effect of Time (Period), and Interaction Effects Between Time (Period) and Group, in the Participants’ WLEIS and ARP Scores from the Results of the Repeated Measures Analysis of Variance for WLEIS and ARP.

Variables	Group	M (S.D.)		The Main Effect of Time (Period)		Time (Period) X Group Interaction (Effect)		Partial *η*^2^
		Pre-Test	Post-Test	Wilk’s Lambda	F	Wilk’s Lambda	F	
WLEIS—SEA	Experiment	3.12 (0.36)	4.04 (0.28)	0.227	920.846 ***	0.269	732.472 ***	0.731
	Control	3.25 (0.46)	3.30 (0.46)					
WLEIS—OEA	Experiment	3.00 (0.38)	3.89 (0.28)	0.241	850.722 ***	0.306	611.942 ***	0.694
	Control	2.99 (0.40)	3.07 (0.45)					
WLEIS—UOE	Experiment	2.25 (0.65)	3.15 (0.26)	0.223	940.771 ***	0.322	569.308 ***	0.678
	Control	2.19 (0.58)	2.30 (0.59)					
WLEIS—ROE	Experiment	3.14 (0.71)	4.04 (0.75)	0.244	835.913 ***	0.294	649.270 ***	0.706
	Control	3.25 (0.68)	3.31 (0.69)					
ARP—Control	Experiment	3.12 (0.51)	3.78 (0.68)	0.472	301.549 ***	0.895	31.549 ***	0.105
	Control	3.24 (0.49)	3.58 (0.70)					
ARP—Origin and Ownership	Experiment	2.35 (0.90)	3.01 (0.96)	0.463	361.149 ***	0.935	26.537 ***	0.101
	Control	2.41 (0.91)	2.75 (1.04)					
ARP—Reach	Experiment	2.53 (0.75)	3.21 (0.85)	0.495	275.209 ***	0.896	31.452 ***	0.104
	Control	2.56 (0.74)	2.89 (0.76)					
ARP—Endurance	Experiment	2.76 (0.58)	3.42 (0.74)	0.469	332.318 ***	0.955	28.724 ***	0.101
	Control	2.75 (0.58)	3.09 (0.79)					

*** *p* < 0.001.

## Data Availability

Not applicable.
